# Proceedings of the Buffalo Medical Association

**Published:** 1860-02

**Authors:** W. H. Butler


ART. III.—*Proceedings of the Buffalo. Medical Association—December Meeting.* Reported by W. H. BUTLER, M. D., Secretary.

PROF. ROCHESTER read the following interesting report of a case of Puerperal Eclampsia—termination fatal.

The extremely interesting account of an instance of recovery from puerperal convulsions, after great prolongation of most alarming symptoms, narrated to the Association, at its last meeting, by Prof. White, induces the presentation of the following case, to which allusion was then made. It is brought forward, not from a controversial or antagonistic disposition, but simply to place on record the failure of all therapeutic measures to prevent a fatal issue, although more timely intimation of danger than usual was given, and was promptly attempted to be averted.

On Tuesday, Aug. 2d, the writer was consulted, at his office, by Mrs. P. She was between seven and eight months advanced in her sixth pregnancy. The writer had attended her in her last two labors, both of which were light, and convalescence rapid. She stated that she had not been well during her present gestation; had experienced excessive anxiety, and had undergone much fatigue; had been constantly threatened with premature labor, and for the last ten days had been very giddy and sick, with paroxysms of intense headache, accompanied with dimness of vision, ringing in the ears, and nausea; her face was constantly flushed, and her features swollen; bowels confined, urine scanty, and highly colored. She was requested to return at once to her residence, and to send some of the urinary secretion for examination. This was found to be very heavily loaded with albumen. Visited her immediately, and took, by cups over the lumbar region, eight ounces of blood; directed rest, cold applications to the head, and two drachms of Rochelle salts to be taken every four hours.

Aug. 3d, 9 A. M. Is something better than yesterday; the stomach, however, is very iritable, and there has been no movement of the bowels. Seidlitz powder every two hours; cold to the head is found grateful, and is ordered to be continued.

2 P. M. Nausea less persistent, bowels have moved once slightly, urinary secretion small. Treatment continued.

6 P. M. Was summoned in haste; found labor-pains had set in about 4 P. M. The membranes had just ruptured on my arrival. The os uteri was fully dilated, and the head had commenced its descent. There was an interval of about ten minutes between the pains. With each pain the face became much flushed, and as it subsided, there was intense headache, ringing in the ears, and nausea. The pulse was firm and full, but natural in frequency. At 7 P. M., took twelve ounces of blood from the arm. The pain in the head was much lessened, and at 8 P. M. the child was born. While the head was pressing upon the perineum, a little chloroform was inhaled, about one drachm altogether. The placenta was discharged into the vagina, spontaneously. After its removal, the flowing was free, but not excessive (the preceding labor had been succeeded by severe uterine hemorrhage). After ascertaining that the uterus was well contracted, administered half a grain of morphine; and at 10 P. M. left the patient very comfortable. The husband and nurse were both enjoined to keep a careful watch.

Aug. 4th, 6 A. M. At this time, just ten hours after delivery, Mrs. P. was suddenly seized with a severe convulsion. She had slept most of the night, and at the moment of her seizure was conversing cheerfully. Before half an hour had elapsed, the writer was at her bed-side. She complained of a slight pain in the head, but was perfectly quiet, and was *not aware* that she had had a convulsion. She complained that her tongue was sore, but was not conscious that she had bitten it severely. The lochial discharge was very slight. No urine had been passed since delivery, and only a small amount was found by catheterism. The pulse was full and firm, and numbered eighty. The face was flushed, and the conjunctivæ injected. V. S. repeated to ten ounces, when symptoms of syncope were evident.

Administered calomel, grs. xij.—directed sinapisms to lower extremity, cold to the head, and enjoined perfect quietude. 9½ A. M. Feels quite easy, no pain in head, lochia more free; administered Rochelle salts, two drachms.

12 M. While the writer was sitting by her side, and just as she had remarked, "that she felt very well," Mrs. P. was seized with a violent convulsion. There was no warning; its access was instantaneous. It lasted for nearly two minutes, and was followed by a heavy stupor; attempted to administer chloroform, but do not think any was inhaled during the paroxysm. As soon as possible directed a large enema, of castor-oil, spirits of turpentine, tr. of assafœtida, and salt water, to be administered. This did not even come away, and in half an hour was repeated: no dejection. At this time the heavy stupor ceased, but Mrs.P., although apparently conscious, could not articulate distinctly. The face became pale, the surface moist, the pulse less firm, but not increased in frequency. The extremities were cool and the scalp hot. Prescribed ol. tiglii, gtt. ij. every hour, ice to the head, dry heat to the surface and extremities: the patient under the continued but gentle use of chloroform.

3 P. M. Prof. White in consultation. Treatment continued.

4 P. M. Another *instantaneous* and severe convulsion; the patient was in the very act of inhaling chloroform, of which three ounces had been used in the preceding three hours. The stupor which supervened was not so profound or long-continued as that which succeeded the second convulsion. The tongue, in spite of every effort at prevention, was badly lacerated. The teeth of the upper jaw were very large and overhanging; Croton oil and chloroform continued, and another purgative enema administered. Brandy and beef-essence directed. 6 P. M. No dejection from the bowels. At the suggestion of Prof. White, croton oil was obtained from another source. The administration of this was followed in less than an hour by a very copious operation. 8½ P. M. The patient remaining in about the same condition, had another convulsion, attended with an enormous watery evacuation from the bowels, but succeeded by no improvement. The inhalation of chloroform was continued; one grain of morphine was administered; the hair was cut close, and the ice-cap applied, and brandy and beef-essence given very freely. Four ounces of urine were removed by catheterism; it was highly albuminous. The tongue was now so much swollen that it remained protruded, and deglutition was difficult. There was neither profound stupor nor paralysis; the pupils were sensitive, but from this time there was probably absence of consciousness. 10 P. M. Another convulsion, followed by a watery involuntary evacuation of the bowels. Prescribed tr. opii ʒj. by enema; ammonia carb., grs. v. every hour; stimulants and beef-essence as freely as possible; a large sinapism over the spine, and a blister, 4 × 4, to the back of the neck. The pulse now numbered 120, and was very feeble. It is unnecessary to give a further detail of the symptoms; the patient continued to fail, having convulsions at irregular intervals, and died half an hour after the last, at 4 P. M., on the 6th. She had, altogether, thirteen convulsions. No post-mortem examination could be obtained.

*Remarks.*—The occurrence of the first convulsion so late as ten hours after delivery, although not unprecedented, is noted as unusual, especially as the threatening symptoms had ceased. The instantaneous accession of the convulsions, without any twitchings or premonitory manifestations of any kind, is not less remarkable. After the second convulsion, the writer scarcely left the bedside until all hope was over; he at once administered chloroform, and for at least twelve hours the patient was kept under its constant influence. It did not appear to have any control over the paroxysms, they coming on in the very act of inhalation. The chloroform was of excellent quality (Duncan & Flockharty's), it was given fearlessly, and, from abundant experience in its use, it may be permitted to add, judiciously and safely.

### DISEASE OF KNEE-JOINT.

Dr. Miner presented a specimen of scrofulous disease of knee-joint, for which he had made amputation that morning at the Buffalo General Hospital. He called the attention of members to the diseased appearance of the cancellated and medullary portion of the bone, together with the numerous large abscesses which had formed in the head of the tibia, and condyles of the femur; and referred to the following history, furnished by Dr. Steel, Resident Physician and Surgeon of the Hospital.

A. C., æt. 24, American, laborer, of temperate habits, was admitted into the Buffalo General Hospital, Nov. 29th.

His right knee was much swollen, tender, and flexed at nearly a right angle, with inability to straighten it.

Opposite the head of the fibula was a small ulcer with an opening, about its center, which was discharging freely a greenish-yellow matter. By introducing a probe upwards and inwards three inches, could be felt the external condyle, roughened by caries.

The patient was emaciated and oligæmic, of fair complexion and sandy-colored hair. His father died of "quick consumption." He first perceived swelling of knee nine months before his admission, but did not attribute its access to any injury. Was able to use limb for five months, after which was incapacitated by pain and stiffness of joint. Swelling continued to increase up to 4 weeks previous to his admission; at which time it was lanced, discharging pus freely. Had had night-sweats and hectic fever with paroxysms of pain, at different times.

Dec. 6th. Patient, being brought under chloroform, suffered amputation (flap method) of right thigh, about six inches above joint. Shortly after the operation, reaction occurred; but for thirty-six hours his pulse continued frequent and feeble; occasional nausea with vomiting.

Dec. 12th. Pulse 92, full and strong, capillary circulation good, appetite moderate, stump doing well, discharging pus freely. Treatment has been quinia, beef-essence, and stimulants.

An examination of the diseased joint disclosed extensive caries of head of tibia and external condyle of femur; there being small cavities in each. All the articulating surfaces were roughened, and the ligaments had disappeared. Purulent and cheesy or tuberculous matter, to the amount of a quart, was infiltrated in and around the joint, and under the muscles next the bones, up the thigh and down the leg, each way three inches. The periosteum was softened and easily separated from the bone (femur); the medullary substance of which being of less than the usual consistency.

Dr. Hamilton introduced to the Society, Dr. William K. Scott, who he said was now in his 72d year, and who was the first physician licensed to practice medicine and surgery by the state censors; his license being the first on the list of that large body of men, who have since been licensed by the same board. He was admitted to practice in the year 1808. He is now a resident of this city, but has for many years withdrawn himself from the active duties of his profession. Dr. Hamilton stated then, he had introduced Dr. Scott to the society for the purpose of calling the attention of the gentlemen to a most extraordinary achievement or feat in penmanship—the most extraordinary he believed upon record—and especially with a view to illustrate to what an extent the organ of sight might be preserved and cultivated, even in advanced age. Dr. Scott had for a long time declined allowing any publicity being given to this fact; but at Dr. Hamilton's urgent solicitation he had finally consented.

Dr. White moved that Dr. Scott be requested to present the specimens and to favor the society with some remarks upon the subject.

Dr. Scott then stated in substance as follows. His object in making these experiments was to benefit and retain his sight. Three years ago his eye-sight was so imperfect that he could not read any ordinary print without the aid of spectacles, as had been the case for many years previous. At that time he had occasion to do some writing so fine as to require the aid of a magnifying glass. After writing at intervals for a few days, he found his eye-sight improved; and knowing the effect of exercise upon all our faculties, concluded to systematically pursue this exercise of the eye, and note the result.

The writing was done upon an enameled card with a metallic point. The glass used was a common pocket lens, with a focal distance of $\frac{3}{4}$ of an inch, which was held in the left hand while writing. In this manner a little was written nearly every day, always stopping before the eyes were fatigued. When what was at first thought to be very fine writing could be easily done, still finer was attempted.

The specimen presented was written last May, at which time the Dr. could read with ease the finest print without spectacles. Since that time this exercise has been omitted, and his sight is not now quite as good as it then was, but he believes that writing a few days will make it as good as ever. He finds it difficult to write in hot weather.

*Description of the Specimens presented.*

Upon a circle, $\frac{57}{100}$ of an inch in diameter, or a minute fraction larger than a three-cent piece, is written :—

| | | |
|---|---|---|
| 1. | The Lord's Prayer . . . . | 66 words |
| 2. | The Apostles' Creed . . . | 109 " |
| 3. | The Parable of the rich man and Lazarus . | 300 " |
| 4. | " of the ten virgins . . | 207 " |
| 5. | " of the barren fig-tree . . | 92 " |
| 6. | The Beatitudes . . . . | 144 " |
| | and five Psalms, namely, | |
| 7. | The first . . . . . | 130 " |
| 8. | The fifteenth . . . . | 100 " |
| 9. | The one hundred and twentieth . . | 88 " |
| 10. | The one hundred and thirty-third . | 69 " |
| 11. | The one hundred and thirty-first . . | 60 " |

In all 1365 words; and with the caption of each, 1391 words. (The Declaration of Independence contains 1326 words.) Each letter is perfectly formed, every *t* is crossed and every *i* is dotted. The lines are at the rate of one hundred and fifty to one inch; there being eighty-five lines in this piece, of which seventy-five are written upon, and ten left blank, as spaces between the subjects.

Dr. Scott showed the society also another specimen, fifty per cent. finer; there being on it 225 lines to one inch, with the Lord's prayer written upon a single line of less than one inch in length.

Drs. White, Miner, Wilcox, Eastman, and several other gentlemen, having examined the specimen, made remarks upon the subject of improving vision by the use of the eye. They were agreed in the opinion that nothing could more conclusively demonstrate the advantage which might in most cases be derived from such practice, both to vision and to the hand, since the steadiness of hand here displayed was a circumstance no less remarkable than the accuracy and perfection of vision. The gentlemen felt very grateful to the Doctor for having consented to bring his experience before them, and they thought such facts might hereafter prove of great practical value to others.

Dr. Rochester said, that he had been very much interested in the remarks of Dr. Scott, and certainly regarded the specimens of caligraphy presented by him, as evidences of vision and of nerve, extraordinary in anybody, and almost miraculous in a person of his years; he begged, however, to suggest to the members of the Association that Dr. Scott, so far from avoiding the use of lenses, had been employing very powerful ones, as all this writing had been performed by the aid of a strong microscope; and that as he had been in the habit of writing and reading microscopically, he experienced no difficulty in reading ordinary print with unaided vision. Dr. Rochester begged leave to state further, that he thought spectacles selected by a skillful optician strengthened rather than weakened the vision, and that persons who were obliged to employ them often after a while laid them aside and found their vision stronger than before.

Dr. Butler remarked that he had worn spectacles for a number of years, and that he believed his eyes had in no way become weakened by their use, that they were really stronger than when he first began to wear them.

